# A gene network engineering platform for lactic acid bacteria

**DOI:** 10.1093/nar/gkv1093

**Published:** 2015-10-25

**Authors:** Wentao Kong, Venkata S. Kapuganti, Ting Lu

**Affiliations:** 1 Department of Bioengineering, University of Illinois at Urbana-Champaign, Urbana, IL 61801, USA; 2 Institute for Genomic Biology, University of Illinois at Urbana-Champaign, Urbana, IL 61801, USA; 3 Department of Chemical and Biomolecular Engineering, University of Illinois at Urbana-Champaign, Urbana, IL 61801, USA; 4 Department of Physics, University of Illinois at Urbana-Champaign, Urbana, IL 61801, USA

## Abstract

Recent developments in synthetic biology have positioned lactic acid bacteria (LAB) as a major class of cellular chassis for applications. To achieve the full potential of LAB, one fundamental prerequisite is the capacity for rapid engineering of complex gene networks, such as natural biosynthetic pathways and multicomponent synthetic circuits, into which cellular functions are encoded. Here, we present a synthetic biology platform for rapid construction and optimization of large-scale gene networks in LAB. The platform involves a copy-controlled shuttle for hosting target networks and two associated strategies that enable efficient genetic editing and phenotypic validation. By using a nisin biosynthesis pathway and its variants as examples, we demonstrated multiplex, continuous editing of small DNA parts, such as ribosome-binding sites, as well as efficient manipulation of large building blocks such as genes and operons. To showcase the platform, we applied it to expand the phenotypic diversity of the nisin pathway by quickly generating a library of 63 pathway variants. We further demonstrated its utility by altering the regulatory topology of the nisin pathway for constitutive bacteriocin biosynthesis. This work demonstrates the feasibility of rapid and advanced engineering of gene networks in LAB, fostering their applications in biomedicine and other areas.

## INTRODUCTION

Lactic acid bacteria (LAB) are a group of Gram-positive, acid-tolerant bacteria closely associated with human life. They are widely used in the fermentation of food products (1), such as cheese and yogurts, and are also attractive cell factories for the production of biorefinery chemicals, including lactic acid, ethanol and others (2,3). Additionally, due to their long history of safe use and natural benefits to human health, LAB serve as promising candidates for therapeutic purposes such as the mucosal delivery of proteins and DNA vaccines (4,5). In the past 15 years, synthetic biology has emerged as a highly promising field for cellular functionality programming (6–14); its rapid movement into the clinic has further positioned LAB as a versatile cellular chassis for biomedical applications (15,16).

To exploit the full potential of LAB, one fundamental need is a powerful capacity for the engineering of complex gene networks, such as biosynthetic pathways and multicomponent artificial gene circuits. One of the underlying reasons is that implementation of cellular functions, including those of LAB, typically requires complex pathways that consist of multiple genetic parts. For example, a 14-gene cluster (15 kb) is involved in *Streptococcus thermophilus* for producing exopolysaccharides that improve fermented milk texture and promote antitumor effects (17); in another case, in *Lactobacillus salivarius*, an 11-kb sequence composed of eighteen open reading frames is responsible for protecting mice from infection by *Listeria monocytogenes* (18,19). Another reason is that, in addition to naturally existing pathways, complex synthetic circuits that consist of multiple parts and modules are often mandatory in order to confer LAB with custom-tailored functionality. Moreover, for both natural and synthetic networks, it often requires systems-level, combinatorial modifications of the entire networks, rather than simple overexpression or knockout of individual genes, in order to achieve desired phenotypes (20,21).

Although basic genetic tools for LAB have been well documented (22–26), the state of art of LAB engineering has primarily remained at a relatively simple level that involves the manipulation of single genes or promoters only. Recently, a couple of new methodologies have been established, including single-stranded DNA (ssDNA) based recombineering (27) and CRISPR–Cas9 assisted recombineering (28), which offer new strategies for manipulating LAB chromosomes. Despite these valuable advances, the engineering of complex LAB gene networks, however, has not been systematically developed, which hampers wide applications of LAB in sophisticated settings. On the other hand, technologies for the engineering of model organisms such as *Escherichia coli* have been significantly advanced over the past few years, shaping the way we program cellular functionality (29–36). Collectively, these facts have motivated us to transform LAB engineering paradigms with new methodologies.

Here, we present a synthetic biology platform for rapid engineering of complex gene networks in LAB. The platform involves a shuttle system and two associated engineering strategies. We first constructed a copy-controlled, broad-host-range shuttle for hosting gene networks. We then examined two strategies that enable rapid editing of both small DNA parts, such as ribosomes binding sites (RBSs), and large building blocks such as individual genes. A complex pathway responsible for nisin biosynthesis was adopted for the demonstration and characterization of the strategies. From paradigms to practice, we demonstrated the platform by applying it to generate a library of nisin pathway variants that have designed translational efficiencies of key genes and corresponding nisin productivities. We further demonstrated the utility of the platform by altering the regulatory topology of the nisin pathway through pathway refactoring, resulting in constitutive bacteriocin biosynthesis.

## MATERIALS AND METHODS

### Strains and growth conditions

Electrocompetent *E. coli* NEB10β (New England BioLabs) was used for general cloning and grown in LB medium at 37°C. *Escherichia coli* EPI300 (Epicentre) was used to induce pCCAMβ1 derived plasmids to high-copy number with addition of CopyControl Induction Solution. A *mutS*-deficient strain *E. coli* NEB10β *mutS*::*amp* was constructed to perform ssDNA recombineering. *Lactococcus lactis* MG1363 was the host strain for the nisin gene cluster and its variants. It was grown at 30°C in M17 broth containing 0.5% (w/v) glucose (GM17). When necessary, the following final concentrations of antibiotics were added: 100 μg/ml ampicillin, 250 μg/ml erythromycin, 150 μg/ml streptomycin, 100 μg/ml spectinomycin, 50 μg/ml kanamycin and 3.5 μg/ml tetracycline. Erythromycin was added at 5 μg/ml when culturing *L. lactis* containing pCCAMβ1 derived plasmids. All the plasmids were first constructed and characterized in *E. coli*, and then transformed into *L. lactis*.

### Platform construction and nisin gene cluster cloning

The Gibson assembly method was used for the construction of plasmids in the study (37). The PCR enzymes and the Gibson reaction enzymes were purchased from New England BioLabs. Unless elsewhere indicated, Q5 High-Fidelity DNA Polymerase was used for amplifying the fragments needed for plasmid assembly and OneTaq 2X Master Mix was used for short fragment (≤ 6 kb) PCR verification. *E. coli* EPI300, pCC1BAC and CopyControl^TM^ induction solution were purchased from Epicentre. The primers used in this study are summarized in Supplementary Table S1.

The PEVLAB system, pCCAMβ1, was constructed as follows: The copy control origin was amplified from the copy-control plasmid, pCC1BAC (38), using the primers, PCCF and PCCR2. The origin of pAMβ1 was amplified from the plasmid pMSP3535 (25) using the primers PAMR1 and PAMF2. These two fragments were then assembled by Gibson assembly and transformed into NEB10β competent cells. The correct plasmid was further transformed into EPI300 to verify if its copy number can be induced by the induction solution (Epicentre).

High-quality genomic DNA of *L. lactis* K29 was extracted using the modified CTAB method (39). The nisin gene cluster was amplified with primers Nisin159/Nisin14703 and by using 200–500 ng genomic DNA as template. The primers, pccamnisR and pccamnisF, were used to amplify the backbone of pCCAMβ1. The 14.5 kb nisin gene cluster and pCCAMβ1 were assembled to generate the plasmid pWK6.

### Strain and plasmid construction for the SPE strategy

To construct the *mutS*-deficient strain for ssDNA recombination, the ampicillin resistant gene (*amp*) was selected to replace the *mutS* gene in the chromosome of NEB10β (suitable for large plasmid and BAC cloning) by Red/ET recombination. Briefly, the *amp* gene was amplified from pUC19 using the primers, MutSAmpF and MutSAmpR. The 5′ ends of the primers contain homologous regions corresponding to the upstream and downstream sequences of the *mutS* gene. Then, gene replacement was performed in NEB10β that harbors pRedET (Gene bridges) by using the standard Red/ET recombination procedure (40). The resulting mutant NEB10β *mutS*::*amp* was verified by PCR with the primers, MutSF and MutSR, and also by sequencing.

The gene fragments of Beta protein, LacI repressor/P_lac_ promoter, pUC origin and *aadA* (spectinomycin resistant gene), were amplified from the plasmids pKD46, pINV5, pUC19 and pTKRED, respectively (40,41). Then, these four fragments were assembled to generate the IPTG inducible high-copy Beta expression plasmid, pBeta. The plasmid was verified by sequencing.

### Small part editing (SPE) by ssDNA-based recombineering

Liquid cell cultures were inoculated from overnight cultures and grown to an OD_600_ of 0.3 in a 37°C shaking incubator. IPTG was added to the cultures at a concentration of 1 mM to induce λ Beta protein expression for 45 min. Cells were collected at 4°C and washed twice with ice cold water. The cell suspension was added with 90-nt oligos, transferred to a prechilled 1 mm gap electroporation cuvette (Bio-Rad) and electroporated at 1.8 kV with an Eppendorf Eporator. The cells were recovered by adding 1 ml of LB immediately and grown at 37°C for 2 h. Further details can be found in Supplementary Materials and Methods.

### Large part editing (LPE) by selection and counter-selection

In the selection step, Red/ET expressing cells (NEB10β/pRedET) that contain pCCAMβ1-derived plasmids were first grown up to an OD_600_ of 0.3 at 30°C. Arabinose was added at a final concentration of 0.4% to induce Red/ET expression at 37°C for 45 min. In a 4°C environment, cells were collected, washed and electroporated with *rpsL-neo* DNA flanked with homologous arms. After recovery at 37°C for 1 h, cells were spread on kanamycin and erythromycin plates at 30°C for selection of *rpsL-neo* allelic replacement. For counter-selection, competent cells of correct recombinants were prepared using the same procedure as described in the selection step. The cells were then electroporated with nonselectable DNA fragments that contain the same flanking homologous arms. The counter-selection transformants were subsequently selected on streptomycin and erythromycin plates at 37°C. Detailed methods are described in Supplementary Materials and Methods.

### Nisin pathway refactoring via topological alteration

Replacement of the nisin-inducible promoters PnisA and PnisF and deletion of *nisRK* were made by two rounds of selection and counter-selection experiments based on the plasmid pWK6. First, *rpsL-neo* DNA with PnisA flanking sequences was used to replace the *nisA* promoter. Then, the PlctA promoter of lacticin 481 was amplified from *L. lactis* CNRZ 481 (42). The PCR product flanked with homologous arms was used as nonselectable DNA to replace *rpsL-neo* under streptomycin selection. The resulting plasmid pWK6-PlctA was further modified by replacing the region of *nisR*, *nisK* and PnisF with *rpsL-neo* DNA. Finally, the *rpsL-neo* DNA was replaced with the PlctF promoter of lacticin 481, generating the plasmid pWK6-PlctA/F. The plasmid was transformed into *L. lactis* MG1363 and nisin productivity was tested using GM17 medium with different pH values: GM17-pH6.7 (original) and GM17-pH6.0 (adjust the initial pH with acetic acid).

### Nisin productivity test

The plasmids containing the nisin gene cluster or its derivatives were first transformed into *L. lactis* MG1363. Colonies were then picked up from the GM17/Erm plate and inoculated to GM17/Erm broth at 30°C for 24 h. For each sample, the culture was transferred to fresh GM17/Erm liquid medium at a ratio of 1:50. Five hundred microliters of culture was taken every 2 h to measure the optical density at 600 nm (OD_600_). Starting from an OD_600_ of 2.0, 500 μl of culture was taken and centrifuged at 10 000 × *g* for 5 min every 2 h for five times. The supernatants were diluted with pH 2.0 HCl/1% Tween-20 by 3-fold, and further used for nisin productivity measurement. The nisin productivity was determined by a double layer agar diffusion method that involves the following steps (43): First, a bottom layer was prepared by mixing 50 μl of an overnight culture of *L. lactis* 117 (44) with 25 ml of molten media agar (precooled to 50°C) and pouring into a 150 mm plate. Half an hour later, the bottom agar was overlaid with an additional 25 ml of molten soft agar. Meanwhile, a treated 96-well PCR plate (well maker) was placed in the upper layer to make wells. Another half an hour later, the PCR plate was removed and 15 μl of standard nisin solution (25–400 IU/ml) or samples were added into wells. Afterward, the plate was incubated at 30°C for 10 h. A standard curve of nisin inhibition zone versus nisin concentration was drawn by measuring the diameters of inhibition zones produced by standard nisin (Sigma-Aldrich). With this curve, the concentrations of nisin from tested samples were estimated. The well maker and nisin standard curve are illustrated in Supplementary Figure S3.

## RESULTS

### Constructing an engineering platform for complex LAB gene networks

**Figure 1.**
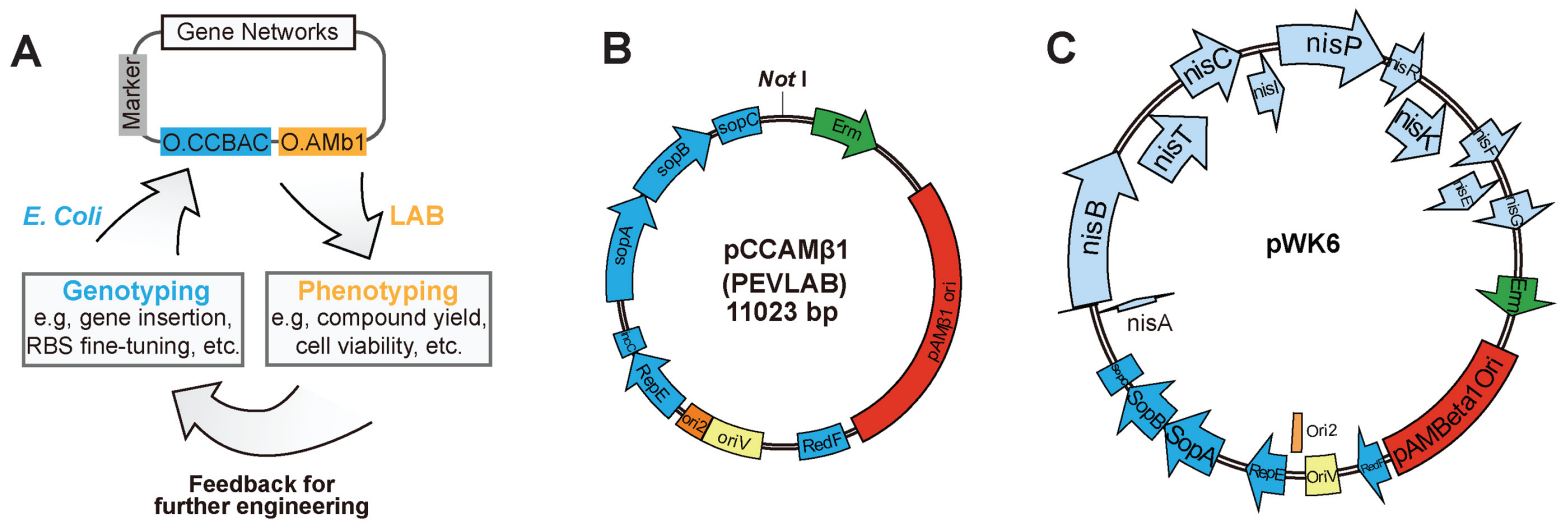
A gene network engineering platform for lactic acid bacteria. (**A**) Schematic of the engineering platform. The platform is based on a copy-controlled shuttle that contains a gene network insertion site, a selection marker and two compatible origins of replication, O.CCBAC and O.AMb1. The former enables copy-controlled replication in *E. coli* for efficient genetic editing while the latter allows broad-host-range replication in LAB for necessary phenotypic testing. Multi-cycle iteration between genotyping and phenotyping enables facilitated engineering of complex LAB gene networks. (**B**) Map of the vector pCCAMβ1. Single-copy replication origin ori2, inducible high-copy origin oriV and lactococcal replicon pAMβ1 are all shown in the map. Here, *SopA*, *SopB* and *SopC* ensure accurate partitioning of the plasmid during cell division; *RepE* encodes the replication initiation protein; incC is the incompatibility region of the bacterial F plasmid; and *RedF* encodes a resolvase. *Not* I is a unique cutter for insertion and plasmid linearization. (**C**) The plasmid pWK6. It is composed of the vector pCCAMβ1 inserted with a 14.5 kb-long nisin biosynthesis pathway.

To enable rapid engineering of gene networks in LAB, we started by creating a shuttle system that allows stable propagation in both LAB and *E. coli*. As shown in Figure 1A, the system consists of a gene network insertion site, a selection marker and two compatible origins of replication, O.CCBAC and O.AMb1. O.CCBAC is a copy-controlled bacterial artificial chromosome (BAC) origin that replicates only in *E. coli* and remains single-copy but can be induced to have high copies when needed (38). O.AMb1 is a broad-host-range origin of replication that functions in multiple LAB species (22). Such a design enables quick manipulation of targeted gene networks by leveraging synthetic biology tools that have proven useful in *E. coli* but may not be directly transformable to LAB. At the same time, the design allows direct phenotypic screening in LAB without the need for additional engineering. We named the system PEVLAB, standing for pathway engineering vehicle for lactic acid bacteria.

One essential feature of the PEVLAB system is its copy number controllability. During genetic manipulation, including both single-stranded DNA-based recombination (45) and Red/ET-based recombineering (46), it is critical to keep the target network single-copy because, otherwise, the efficiency of editing will be significantly reduced and, as a result, recombination will become unfeasible. Here, the copy-controlled feature of the system enables a target network to remain as a single copy, therefore enabling high-efficient homologous recombination. In the meanwhile, it is necessary to have a high copy number for a target gene network during DNA extraction. Again, the copy-controlled feature of the system can facilitate the harvest of the gene network by increasing its copy number when needed. Although there have been several reported shuttle systems that propagate in both LAB and *E. coli* (22,47–50), our vehicle is the first system conferring copy number control that is critical for efficient pathway editing.

To develop PEVLAB, we first constructed a vector plasmid, pCCAMβ1 (Figure 1B), using the Gibson assembly method and standard recombinant DNA techniques (Section Materials and Methods). To demonstrate the shuttling feature, we subsequently cloned into the vector a complete pathway for the biosynthesis of nisin (51), an antibacterial peptide that is commonly used in the food industry as a natural preservative. The pathway consists of 11 genes within four operons that are encoded in 14.5 kb of DNA; it also contains a complex genetic architecture with internal feedback regulation (52,53). The pathway thereby represents a showcase of complex gene networks in LAB. Although it is one of the most well-known LAB pathways and has wide applications in industry, its large size and complicated architecture create a great challenge for pathway construction and optimization. Up to date, there is no report on systematic, large-scale manipulation of the pathway. Therefore, for our purpose, the nisin pathway serves as a perfect example for developing and demonstrating the platform. Upon construction, we found that the resulting plasmid, pWK6 (Figure 1C), enables heterologous production of bioactive nisin in a *Lactococcus lactis* strain (MG1363) (Supplementary Figure S2), which demonstrates successful construction of the PEVLAB system.

### Multiplex and continuous editing of small DNA parts

To achieve desired functionalities, systematic, multicycle optimization is often required for both natural and engineered gene networks. The underlying reason is that natural networks typically need global optimizations for a better performance while synthetic networks demand tedious fine-tuning to obtain designed goals. Meanwhile, when considering the building blocks that constitute gene networks, there are primarily two classes—small DNA fragments, such as ribosome-binding sites (RBS), promoters and spacers and large DNA parts such as genes and operons. Therefore, to facilitate the engineering of complex gene networks in LAB, we proceeded to develop strategies for efficient editing of different sizes of building blocks by leveraging the unique feature of the PEVLAB system.

**Figure 2.**
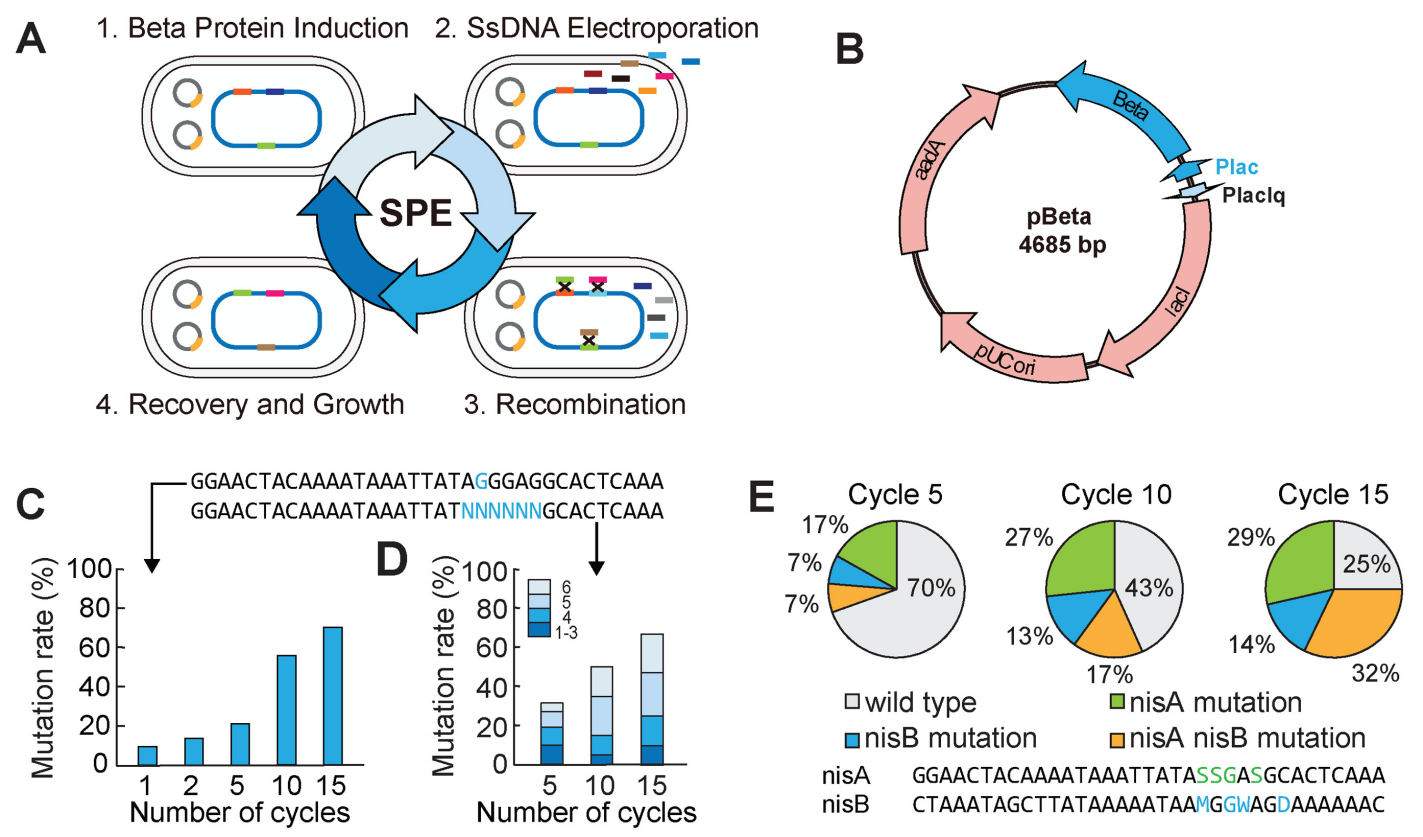
A strategy for rapid editing of small DNA parts. (**A**) Detailed workflow for the strategy that empowers continuous and multiplex small part editing (SPE). The strategy involves four cyclic steps: (1) induction of the ssDNA-binding protein, Beta protein, in a modified strain that harbors a circuit-containing PEVLAB; (2) electroporation of ssDNA with desired sequences and flanking homologous ends; (3) sequence replacement at the target sites in PEVLAB; and (4) recovery and growth of the resulting cells from electroporation and recombination. (**B**) Map of the helper plasmid, pBeta, which produces Beta protein protecting single-stranded DNA. Beta protein is controlled by the promoter, Plac, which is repressed by lacI and induced by IPTG. The gene *aadA* encodes resistance to spectinomycin. (**C** and **D**) Efficiency of the SPE strategy as a function of cycle number for the modification of single (C) and multiple (D) nucleotides. In both cases, the efficiency increases with cycle number. The sequences of the corresponding oligonucleotides are shown above the panels. (**E**) Multiplex and continuous modification of two small DNA parts (the RBS sequences of *nisA* and *nisB*). Again, the SPE efficiency increases with cycle number.

For small DNA fragments, we proposed to adopt a multiplex, continuous engineering strategy based on the concept of multiplex automated genome engineering (MAGE) (30). As illustrated in Figure 2A, the strategy for small part editing (SPE) starts with the induction of the single-stranded DNA (ssDNA) binding protein, Beta protein (Figure 2B), in our *mutS*-knockout *E. coli* strain (NEB10β *mutS*::*amp*) that harbors a circuit-containing PEVLAB (Step 1); electroporation is then performed to introduce ssDNAs that are composed of desired sequences and flanking homologous end regions (Step 2); facilitated by Beta protein, sequence replacement occurs through DNA replication (lagging strand replacement) at the target sites (Step 3); and, finally, the resulting cells recover from electroporation and recombination and grow up to a density ready for the next round of editing (Step 4). Instead of using the host strains that express the complete set of the λ Red Exo Beta and Gam recombination proteins (30,54), we constructed a new host strain, NEB10β *mutS*::*amp* (recA^−^, endA^−^ and mutS^−^), and paired it with the expression of Beta protein alone. The underlying reason is that NEB10β is known to be appropriate for stable host of large plasmids such as BACs and eliminating recombinase and endonuclease can reduce unwanted recombination.

Importantly, the above procedure can be repeated to empower continuous part engineering and can also be multiplex for simultaneous modification of multiple targets. Notably, the copy-controlled nature of PEVLAB confers efficient multiplex recombination by keeping the system single-copy and simultaneously facilitates plasmid harvest and functional test by switching it to multicopy through induction when needed.

Upon parameter optimizations, we characterized the efficiency of the SPE strategy by using the RBS of the nisin precursor gene *nisA* as an illustrating target. We found that the editing efficiency increases with cycle number (Figure 2C and D). It achieves 70.0% for a single nucleotide change (Figure 2C) and 66.9% for a replacement of six consecutive nucleotides (Figure 2D).

In addition to single part replacement, we also examined the feasibility for multiple part modifications. Figure 2E shows the result from simultaneous modification of two separate targets (*nisA* and *nisB*), suggesting an increasing editing efficiency with cycle number from a total of 30.5% in Cycle 5 to 75.0% in Cycle 15.

### Efficient manipulation of large building blocks

**Figure 3.**
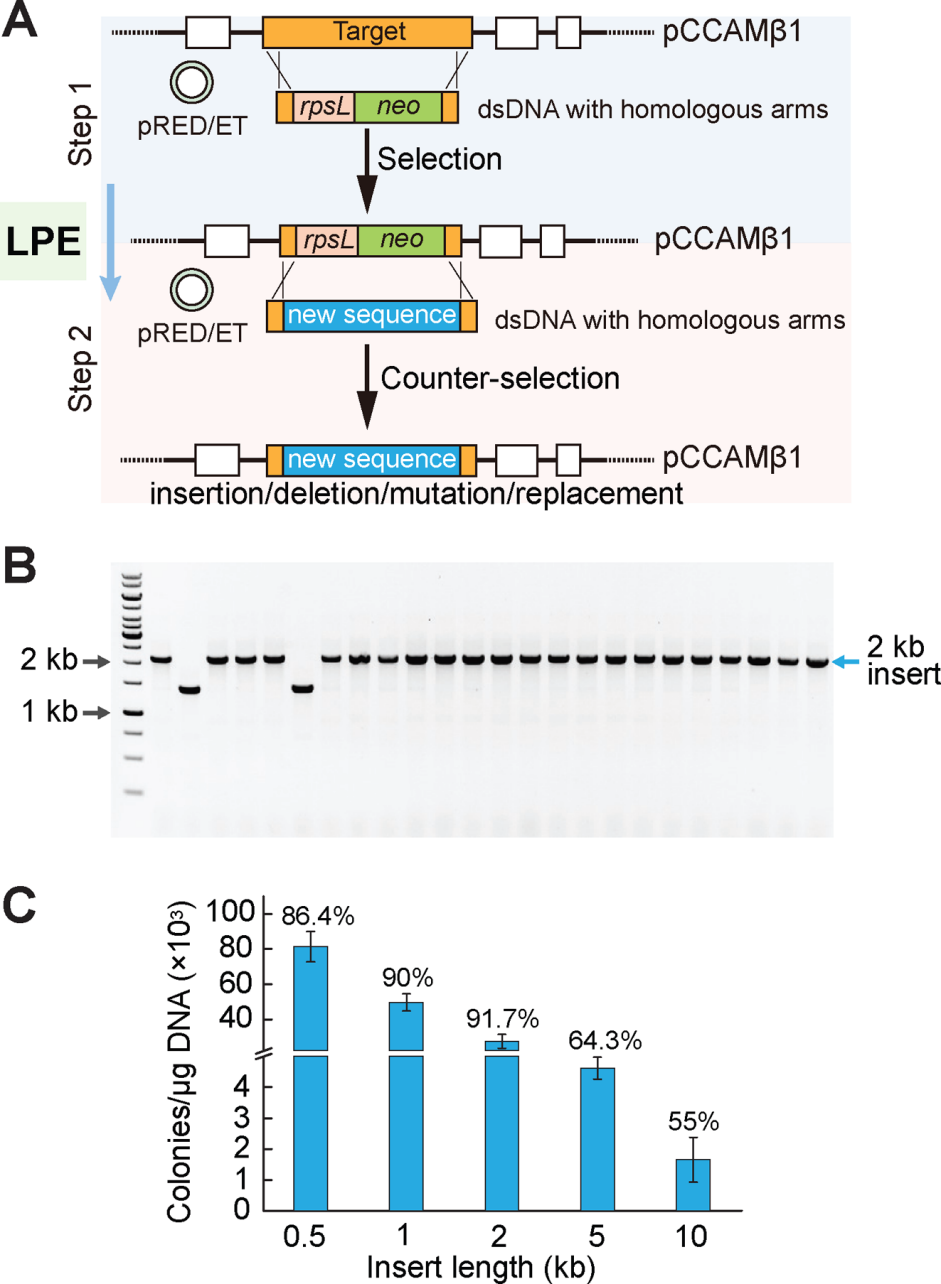
A strategy for efficient manipulation of large building blocks. (**A**) Schematic of the two-step strategy for large part editing (LPE). In Step 1, an *rpsL-neo* selection and counter-selection cassette is first amplified by PCR to contain homologous ends and then introduced into PEVLAB through recombination upon the induction of the Red/ET system. This is followed by transformation and selection on kanamycin. In Step 2, replacement of the *rpsL-neo* cassette occurs by a double-stranded DNA fragment that contains a sequence of interest. Induction of recombination is followed by transformation and selection on streptomycin and erythromycin. (**B**) PCR confirmation of the LPE strategy for replacing a target region with a 2 kb fragment. Eighteen out of twenty samples show correct sizes. (**C**) Efficiency and accuracy of the LPE strategy as a function of insert size.

Although effective in manipulating small DNA parts, ssDNA-based allele modifications are not feasible for large fragments such as genes and operons, due to the difficulty in generating long ssDNA and the inefficiency of recombination with long ssDNA. To circumvent this obstacle, we proposed a large part editing (LPE) strategy by combining the Red/ET recombineering technique with the selection and counter-selection method (55). As illustrated in Figure 3A, this strategy involves two steps. In the first step, a dual selection and counter-selection cassette (*rpsL*-*neo*) is amplified by PCR to contain homologous ends and then introduced into cells to replace the targets in PEVLAB through Red/ET-based recombination. This is followed by selection on kanamycin. In the second step, the cassette is replaced by exogenous DNA fragments that contain desired sequences, again through Red/ET recombination. Counter-selection follows as a result of the toxicity produced by the counter-selectable gene (*rpsL*) under specific conditions.

To test the LPE strategy, we aimed to modify the pathway-containing PEVLAB (pWK6) by replacing a chunk of the DNA by fragments with various sizes. To implement recombination, a pair of ∼42 bp homologous arms was designed for both selection and counter-selection. In addition, in the second step (counter-selection), fragments with a size from 0.5 to 10 kb were introduced as nonselectable DNA for replacing the *rspL-neo* cassette. Notably, the DNA fragments were amplified from the chromosome of *L. lactis* MG1363; therefore, there is no similarity between the fragments and pWK6 plasmid.

Our results showed that the target can be successfully substituted by all of the fragments we tested (Figure 3B and Supplementary Figure S5B–E). To systematically characterize the strategy, we further calculated its efficiency by counting resulting colonies per μg of introduced DNA, and validated their accuracy through PCR (Figure 3C). Interestingly, we found that, although colony number drops for more than 1.5 orders of magnitude with increasing insert size (from 0.5 to 10 kb), the corresponding accuracy remains reasonably high (from ∼90% to 55%).

It is worthy of note that, in counter-selection experiments, the plates spread with cells transformed with nonselectable DNA often appear as a thin layer of resistant cells and the positive recombinants grow on this layer as larger colonies (Supplementary Figure S5A). To facilitate the selection of positive colonies, we reduced the background layer through several strategies: First, fewer cells were spread on streptomycin plates. Generally, 20 μl of cells (1 h of recovery time) produces enough colonies and a weaker background. In our efficiency calculation experiments, reactions with the 0.5–2 kb fragments were found to have a very high rate (Figure 3B). As a result, 0.5–5 μl cells were sufficient to get single colonies on the plate without having a high background level. Second, the plates were incubated at 37°C for 14 h. The underlying reason is that 14 h is long enough to allow the positive recombinants to form the visible colonies; on the other hand, although the positive cells grow faster than the background cells, the background cells will accumulate when given a longer incubation time (>14 h), making it difficult for picking up correct single colonies. Third, although the streptomycin is generally used at a final concentration of 50 μg/ml, we increased it to 150 μg/ml to weaken the background.

It is also worth mentioning that different PCR strategies were used for verification based on the size of inserts: For small inserts (up to 5 kb in size), we used cells of 1 μl of culture, grown directly from colonies, to perform PCR reaction (Onetaq, NEB); for longer inserts such as the 10 kb fragment, we prepared plasmids from the cell culture and used the plasmid as template for PCR reaction (Q5 (NEB)). In some cases, the colony PCR results in two bands, with one corresponding to the desired size and the other corresponding to *rspL-neo* (Supplementary Figure S5B–E), attributed to the contamination by the background cells when picking up colonies. However, the negative band (*rspL-neo*) usually disappears using culture upon two additional rounds of inoculations.

### Case study 1: Generating a library of designed nisin pathway variants

To demonstrate the power of our platform, we sought to apply it to expand the phenotypic diversity of the nisin pathway by creating a library of forward-engineered pathway variants that give rise to distinct nisin productivities. Here, we proposed to achieve the goal by systematically altering the RBS sequences of the genes *nisA* and *nisB*, motivated by the facts that both the former, responsible for nisin precursor production and the latter, responsible for nisin modification, are critical for nisin biosynthesis.

To create variants with altered RBS strengths for *nisA* and *nisB*, we started by designing the translation initiation rate of the RBSs using the RBS Calculator V1.1 (56), a computational RBS prediction tool. During computational design, the ‘Search Mode’ optimization of the RBS Library Calculator was used; 16-nt presequence, 35-nt RBS sequence and 80-nt coding sequence were also used. The resulting RBS sequences were then embraced in a 90-nt ssDNA oligo that is complementary to the lagging strand template for recombination, with the details of oligo sequences and theoretical RBS strengths are listed in Supplementary Tables S2 and S3. To construct the strain library, multiple rounds of recombination were implemented in *E. coli* NEB10β *mutS*::*amp* in order to generate desired RBS mutations. The resulting mixed plasmids were transformed into *L. lactis* MG1363, resulting in a library of nisin-producing strains that contain different designed RBS sequences.

**Figure 4.**
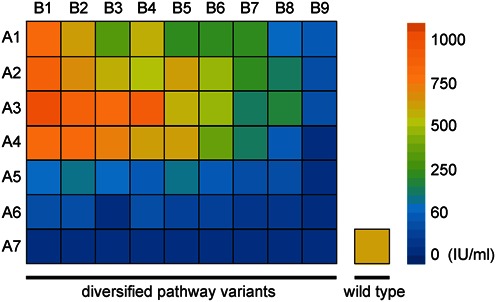
Productivity heat map of 63 forward-engineered nisin pathway variants. A library of phenotypically divergent nisin pathway variants (63 strains) was rapidly generated by simultaneously varying the RBS sequences of *nisA* and *nisB* using the PEVLAB system and associated SPE strategy. The theoretical translation initiation rates of A1–A7 are: 125 591, 46 662, 25 607, 18,134, 13 843, 9233 and 3430, accordingly. Those of B1–B9 are: 186 955, 111 924, 63 941, 54 475, 24 407, 8228, 2197, 798 and 652. The color of the heat map indicates the nisin productivity of the pathway variants measured using a modified agar diffusion assay. The nisin productivity of the wild type is also indicated for comparison.

To quantify the library's nisin productivity, we employed a high-throughput, modified agar diffusion assay (Supplementary Figure S3). Lactococcal strains with different nisin productivity were selected and their plasmids were sequenced. A library of 63 strains with mutant plasmid was created. We found that the resulting strains indeed showed a high degree of phenotypic diversity—their nisin productivity spans from 0 to over 1000 international units (IU) per ml (Figure 4). In addition, a subset of the variants were capable of producing nisin at a yield (maximum 1023 IU/ml) higher than that of the wild-type strain MG1363/pWK6 (600 IU/ml) (Supplementary Table S4). Therefore, this example demonstrated the feasibility of systematic engineering of complex gene networks in LAB with our platform.

### Case study 2: Altering the regulatory topology for nisin biosynthesis via pathway refactoring

**Figure 5.**
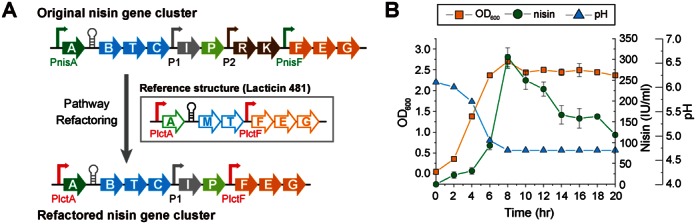
Altering the regulatory topology for nisin biosynthesis by pathway refactoring. (**A**) Schematic representation of the original nisin gene cluster, lacticin 481 gene cluster and restructured nisin gene cluster. Genes with similar function were labeled with the same colors. PnisA and PnisF (green) are nisin inducible promoters. The promoters P1 and P2 in the nisin gene cluster are constitutive. PlctA and PlctF are constitutive promoters and PlctA can be further activated by low pH. After refactoring, PnisA and PnisF were replaced with PlctA and PlctF, and the cassette P2-nisRK was deleted. (**B**) Nisin production profiles of *L. lactis* MG1363/pWK6-lctA/F at GM17 (pH6) medium.

To further illustrate the utility of the platform, we applied it to alter the gene regulatory architecture of the nisin pathway that controls the mode of nisin production. The wild-type pathway (Figure 5A, top row) consists of 11 genes that constitute five distinct modules for precursor production (nisA), transportation and modification (nisBTC), signal peptide cleavage (nisP), immunity (nisI and nisFEG) and signal transduction (nisRK), respectively (51). The biosynthesis is auto-regulated by extracellular nisin, following a density-dependent, quorum sensing manner: Serving as the peptide pheromone, nisin triggers the production of the pathway genes via the promoters PnisA and PnisF through the two-component system nisRK, which in turn leads to the production of mature nisin (57). Due to the complexity of the nisin pathway and the lack of effective genetic tools, there is no alteration of this complex positive feedback loop that has been achieved.

Using the large editing strategy, we started by introducing a copy of previously reported constitutive promoter, P3a, at the upstream of nisin gene cluster and simultaneously removing the signaling module, nisRK. However, the nisin productivity of the resulting pathway was fully abolished (Data not shown), suggesting that the nisin pathway is organized delicately and sensitive to structural changes. Then, we noticed that there are naturally existing bacteriocin pathways in LAB whose biosynthesis is constitutive. One such example is that of lacticin 481, a lantibiotic produced by *Lactococcus lactis* (58,59). As shown in Figure 5A (middle row), the pathway consists of six genes-*lctAMTFEG* (58,59), where the gene *lctA* encodes the precursor peptide, the genes *lctMT* encode transporter and the genes *lctFEG* are responsible for synthesizing immunity proteins. All of these genes have their counterparts, *nisA*, *nisBTC* and *nisFEG*, in the nisin cluster. However, it does not involve quorum sensing module (*nisRK*), peptide precursor cleavage enzyme (*nisP*) and an additional immunity protein (*nisI*). Additionally, the six genes were driven by two constitutive promoters, PlctA and PlctF, although the strength of the former is shown to be pH dependent (60). Inspired by the structural similarity of the lacticin 481 gene cluster and the nisin pathway and encouraged by the functional similarity of the individual genes, we attempted to reorganize the nisin pathway into the structure same as the lacticin 481 cluster for constitutive nisin production. Again, using the large part editing strategy, we deleted the signaling component (*nisRK*) and replaced the nisin-inducible promoters, PnisA and PnisF, with the constitutive promoters PlctA and PlctF of lacticin 481. By transforming the modified plasmid pWK6-PlctA/F (Figure 5A, bottom row) to the host *L. lactis* MG1363, we found that nisin was successfully produced (Supplementary Figure S6). We noticed that the productivity of the restructured cluster (179 IU/ml) is lower than that of the original pathway (600 IU/ml), which is probably due to the fact that the nisin inducible promoter PnisA is very strong with the induction factor being over 1000 (57). Additionally, as PlctA is shown to be activated at low pH, we examined whether low pH increases nisin productivity. The results showed that, when the initial pH dropped from 6.7 to 6.0 (Figure 5B), the nisin productivity was increased to 300 IU/ml, indicating that a lower pH indeed induces the nisin yield as lacticin 481 (60). Notably, the restructured pathway involves neither nisin-inducible gene expression nor the signaling cassette *nisRK*. Therefore, the results indeed demonstrated that the nisin pathway has been reconstructed for constitutive nisin production. To our knowledge, this is the first example that shows the rewiring of regulatory architecture of the nisin pathway for altered bacteriocin biosynthesis.

This case study is complementary to the previous case study because the former demonstrated the large part editing strategy while the latter utilized the small part editing approach. Collectively, the two examples demonstrated the potential of our platform for efficient engineering complex gene networks in LAB.

## DISCUSSION

Like other organisms, LAB often implement useful cellular functions through complex gene networks, such as natural biosynthetic pathways and multicomponent synthetic circuits. Therefore, toward to realize the full potential of LAB, it is essential to acquire a capability for rapid construction, optimization and testing of complex gene networks. In this paper, by using a nisin pathway and its variants as examples, our synthetic biology platform has been demonstrated as a solution to this need. To our knowledge, this is the first example of systematic, large-scale engineering of complex gene networks in LAB.

Notably, our platform is complementary to recent developments of ssDNA-based recombineering in LAB (27,28). In the work by van Pijkeren and colleagues (27,28), two new strategies were developed to manipulate LAB chromosomes, owing to the facts that the both approaches were based on ssDNA-based recombination and that the chromosomes remain mostly single-copy. In addition, due to the size limit of ssDNA (typically below 100 nucleotides), their strategies are more powerful for modifying small DNA parts (e.g., RBS and spacers) but not large fragments such as genes and operons. In contrast, our platform targets on heterologous biosynthesis pathways and large synthetic circuits. Additionally, conferred by the copy-control feature of PEVLAB, our platform enables efficiently engineering of both small DNA fragments, through ssDNA-based recombineering, and large building blocks, using the RED/ET-based recombineering. Moreover, in addition to ssDNA and RED/ET recombination, our platform shall enable the adoption of other available genome editing strategies in *E. coli* for the construction of LAB gene networks. Last but not least, although not examined in our study, the platform shall be highly feasible for pathway engineering for other Gram-positive bacteria, owing to the broad-host-range feature of the origin of replication pAMβ1. It is also important to notice that complete optimization of complex pathways requires not only the manipulation of the pathways but also the fine-tuning of the host. Toward this end, one potential solution that broadens the degree of optimization is to combine our platform with the above approaches that focus on the chromosomes (27,28). From this perspective, our platform will thus serve as an integral part of the LAB arsenals that facilitate deep optimizations.

Our platform can be used for systematic optimization of naturally existing pathways, such as the nisin pathway illustrated in the study; it can also serve as a versatile platform for bottom-up engineering of fully synthetic circuits, including the refactoring of functional gene clusters (61,62). The feasibility of manipulating complex gene networks offers a variety of utilities for this approach. It empowers metabolic engineering for maximizing the end products of functional biosynthetic pathways. It is also well suited for developing next-generation foods by introducing new flavor and nutrition. Toward therapeutic applications, the platform promotes disease treatment and prevention by accelerating the development of designer probiotics. In addition to practical applications, the platform may also be used to address basic biological questions, particularly those relating to the architecture and function of gene regulatory networks (31,63,64).

In summary, this work offers a new solution to the engineering of complex gene networks in LAB. It enables a wide spectrum of LAB-based applications, promising a new angle for synthetic biology in its application age. The work may also aid in the fundamentals of synthetic biology, by facilitating its shift from the engineering of individual parts and modules to the programming of complex networks.

## Supplementary Material

SUPPLEMENTARY DATA
