# Supplementary material for: A gene network engineering platform for lactic acid bacteria
Source: Nucleic Acids Res. 2015 Oct 25;44(4):e37. doi: 10.1093/nar/gkv1093 (PMC4770204; doi:10.1093/nar/gkv1093)
Supplement: SUPPLEMENTARY DATA [file supp_44_4_e37__index.html]

A gene network engineering platform for lactic acid bacteria — SUPPLEMENTARY DATA 

# A gene network engineering platform for lactic acid bacteria

## SUPPLEMENTARY DATA

- SUPPLEMENTARY DATA
