# Supplementary material for: A gene network engineering platform for lactic acid bacteria
Source: Nucleic Acids Res. 2015 Oct 25;44(4):e37. doi: 10.1093/nar/gkv1093 (PMC4770204; doi:10.1093/nar/gkv1093)
Supplement: SUPPLEMENTARY DATA [file supp_gkv1093_nar-01205-met-h-2015-File007.pdf]

Supplementary Data

A Gene Network Engineering Platform for Lactic Acid Bacteria

Wentao Kong[1,2], Venkata S. Kapuganti[3] and Ting Lu[1,2,4]

[1]Department of Bioengineering, [2]Institute for Genomic Biology, [3]Department of Chemical and Biomolecular Engineering, and [4]Department of Physics, University of Illinois at Urbana-Champaign, Urbana, Illinois, USA

Correspondence should be addressed to Ting Lu (luting@illinois.edu)

Table of Contents

Supplementary Materials and Methods (Page 3-5)

Supplementary Discussion (Page 6)

Figure S1: Transformation efficiencies of PEVLAB plasmids (Page 7)

Figure S2: Confirmation of the nisin production by the engineered strains (Page 8)

Figure S3: Schematic diagram of agar diffusion assay and standard curve (Page 9)

Figure S4: Representative temporal nisin production profiles (Page 10)

Figure S5: Counter-selection plate and PCR verification (Page 11)

Figure S6: Nisin production profile of *L. lactis* MG1363/pWK6-lctA/F (Page 12)

Table S1: Summary of primers used in this study (Page 13-15)

Table S2: Sequences of ssDNA oligos used for SPE in this study (Page 16)

Table S3: RBS sequences and their theoretical translation initiation rates (Page 17-18)

Table S4: Mutants with increased nisin productivity generated by modifying RBS of nisA and nisB (Page 19)

Reference: (Page 20)

Supplementary Materials and Methods

Multi-cycle ssDNA recombination for SPE. Ninety-nucleotide (nt) oligonucleotides (oligos) were used for ssDNA mediated allelic replacements (SPE) in this study. All oligos were synthesized by Integrated DNA technologies with standard desalting purification. The first two nucleotides at the 5'-end of the oligos were phosphorothioated to inhibit the exonuclease degradation and increase the recombination efficiency (1). The sequences of the oligos were designed to be complementary to the template of the lagging strand of the replication fork during the pWK6 replication (2). The lagging strand was determined by its distance to ori2, the replication origin of the pCC1BAC. The mismatch bases were located centrally in oligos. All of the oligos used in this study are summarized in Table S2.

Each round of ssDNA recombination was performed as follows: A single colony of NEB10β *mutS::amp*/ pWK6/pBeta was picked up and inoculated into 5 ml of LB/Spec/Erm/Amp medium in a shaking incubator at 37°C overnight. Then 300 µl of the overnight culture was inoculated into a flask with 15 ml of fresh LB/Spec/Erm and incubated at 37 °C for approximately 2 hours. When the OD_{600} reached a value between 0.2-0.3, 150 µl of 0.1M IPTG was added to the culture to induce Beta expression. After 45-60 min of incubation, the culture was transferred to a 50 ml falcon tube and put on ice for 10 min, and then the culture was centrifuged at 3,000 g for 3 min at 4 °C. The cell pellets were washed twice with 30 ml of ice cold water and resuspended with 50 µl of cold water. The cell suspension was added with 2-4 µl of the 100 µM ssDNA, and transferred to a 1 mm gap GenePulser cuvette (Biorad), followed by electroporation at 1,800 V using the Eppendorf Eporator. One milliliter of LB broth was added to the cuvette immediately, and cells were transferred to a test tube and recovered at 37 °C for two hours. The culture can then be used for the next round of ssDNA recombination.

To determine the mutation rate, the plasmids were extracted from the cultures at cycles 1, 2, 5, 10 and 15, and subsequently transformed into *E. coli* EPI300. A total of one hundred colonies from each EPI300 plate were picked up and inoculated into the LB medium containing Erm and CopyControl™ induction solution. After incubating at 37 °C overnight, the plasmids were extracted from the culture and sequenced. The mutation rates were subsequently calculated from the percentage of mutant plasmids.

Calculation of the efficiency and accuracy of counter-selection during LPE. Selection and counter-selection are enabled by Red/ET recombination using the kanamycin resistant gene *neo* and the streptomycin sensitive gene *rpsL* (3). The *rpsL-neo* cassette flanked with homologous arms was first transformed to Red/ET expressing cells (NEB10β/pRedET). The subsequent replacement event was screened on a kanamycin plate and verified by PCR. Afterwards, non-selectable DNA fragments with desired sequences were transformed into the cells carrying *rspL-neo* for another Red/ET based recombination. Streptomycin was used to counter select the cells without *rspL-neo*. The detailed selection and counter-selection procedure is described as follows: A single colony of NEB10β/pWK6/pRED/ET was picked up and transferred to 5 ml of LB /Erm/Tet medium and incubated in

a shaking incubator at 30 °C overnight. Then the overnight culture was inoculated into a 50 ml falcon tube with 5 ml of fresh LB /Erm/Tet at a 1:50 dilution, and incubated at 30 °C. When the OD_{600} reached 0.2-0.3, 100 µl of 20% Arabinose was added to the culture to induce RED/ET expression at 37 °C for 1 h. Then the culture was put on ice for 10 min, centrifuged at 3,000 g at 4 °C for 3 min, washed twice with ice cold water and resuspended with 50 µl of ice cold water. The cells were mixed with 200 ng of DNA (*rspL-neo* PCR product with flanking homology regions) and transferred to a 1 mm gap GenePulser cuvette (Biorad), and then electroporated at 1,800 V using the Eppendorf Eporator. The cells were added with 1 ml of LB broth immediately and incubated at 30 °C for two hours. Then 100 µl of the cells was spread on a Kan/Erm/Tet plate and incubated at 30 °C for 20 hours. Colonies from the plate were picked up and verified by colony PCR. The *rspL-neo* gene cassette in the correct construct was then replaced by non-selectable DNA fragments. The competent cells made by the method above were used in transformations with 200 ng-1 µg non-selectable DNA inserts. Then, the cells were spread on a LB/Erm/Strep plate. The colonies were picked up and verified by PCR.

To calculate the efficiency and accuracy of counter-selection, the loop site between nisA and nisB in the plasmid pWK6 was chosen as the target for DNA replacement. The primers, loopFSmKn and loopRSmKn, were designed and used to amplify the rspL-neo cassette. In addition, the 5'-ends of these two primers contain regions homologous to the upstream and downstream sequences of the loop respectively. The PCR product was then used to replace the loop sequence to generate pWK6-loop::KmStrep. In the second step of *rspL-neo* replacement, non-selectable DNA fragments with different lengths were generated, including sizes of 0.5 kb, 1 kb, 2 kb, 5 kb, and 10 kb. The primers, loopF321F/loopR820R, loopF321F/loopR1324R, loopF321F/loopR2368R, and loopF321F/loopR5210R, were used for amplifying the 0.5 kb, 1 kb, 2 kb, and 5 kb DNA fragments from the genomic DNA of *L. lactis* MG1363 (Genome locus from 2066681 to 2076421). All of these primers contain ~18 matching bases at the 3'-end for template amplification and ~42 bases homologous to the loop structure for allelic replacement. Because the 18-base matching region at the 3'-end is too short to amplify the 10 kb fragment from the genomic DNA, we constructed a plasmid that contains the 10 kb fragment flanked by two 42-base long homologous regions so that the entire primers can match with the template for amplification. Specifically, the primer pairs, LoopF321F/5404R and 5312F/loopR10071R, were used to amplify the first and second 5 kb fragments from the genome of MG1363. The primers looppucF and looppucR were used to amplify the pUC19 plasmid. These three resulting fragments were then assembled to generate the plasmid pUC-10kb, which contains a 10 kb fragment and the two flanking 42-base homologous regions. Afterwards, the primers, loopF321F and loop10071R, were used to amplify the entire10 kb fragment by using the pUC-10kb plasmid as a template. Finally, 200 ng of the 0.5 kb fragment, 400 ng of the 1 kb fragment, 400 ng of the 2 kb fragment, 750 ng of the 5 kb fragment, and 1 µg of the 10 kb fragment were transformed to pWK-loop::KmStrep separately, and 20 µl of each culture was spread on a LB/Erm/Strep plate. The colonies were picked up and the primers, loop-LF and loop-RR, were used to verify both the lengths and sequences of the inserts. The selection and counter-selection efficiency was

calculated by counting colonies per µg of DNA. Accuracy was obtained by calculating the percentage of correct recombination in the colonies.

Supplementary Discussion

Transformation efficiencies of PEVLAB plasmids. One major application of the platform is the construction of mutant libraries for searching for optimal variants. Thus, it is very important that the large plasmid has a high transformation efficiency. We tested the transformation rate of pWK6 (pCCAMβ1 harbors 14.5-kb nisin gene cluster) in *L. lactis* MG1363. The protocol of making competent cells and electroporation used as described in the literature (4). We found an efficiency of ~10^6 CFU/μg DNA for pWK6 in *L. lactis* MG1363 (Figure S1). Although the efficiency of pWK6 is 20 times lower than the widely used pSH71 origin plasmid-pLeiss:Nuc (3.8 Kb) (5), it is sufficient for the library construction and screening.

Nisin productivity measurement. Nisin biosynthesis increases with the growth of cells and stops at stationary phase, with its production peak found at the late exponential phase or early stationary phase of cell growth (Figure S4). As shown in the figure, the strains $A_{25607}B_{8228}$ and $A_{25607}B_{2197}$ reached their production peaks at the onset of stationary phase. The strains that have a relatively higher productivity, such as $A_{25607}B_{186955}$ and $A_{25607}B_{111924}$, reached their peaks two hours after they entered stationary phase. The same results were observed in our other engineered strains as well for most cases (data not shown). We think that this delay is due to the time needed for the high level of intracellular nisin to be processed and exported to the milieu. As a result, the nisin productivities of engineered strains in our study were given in the form of the maximal values of the strains' nisin production curves.

To experimentally determine the maximum, the nisin levels of a culture were measured using the agar diffusion assay for ten hours at intervals of two hours beginning when the culture's OD_{600} reaches 2.0. The highest nisin production level of these samples was chosen as the nisin production level of this strain. The reason we performed five measurements is that it typically takes less than four hours for a culture to enter the stationary phase when the OD_{600} reaches 2.0 and therefore ten hours are sufficient to cover this period. Notably, the extracellular nisin level decreases over time after the peak level is reached in most engineered strains, which was also reported in other research (6-8). This is assumed to be attributed to the absorption of hydrophobic nisin peptide by cells and tubes and degradation by proteolytic enzymes released from the autolysis of stationary phase cells (9,10).

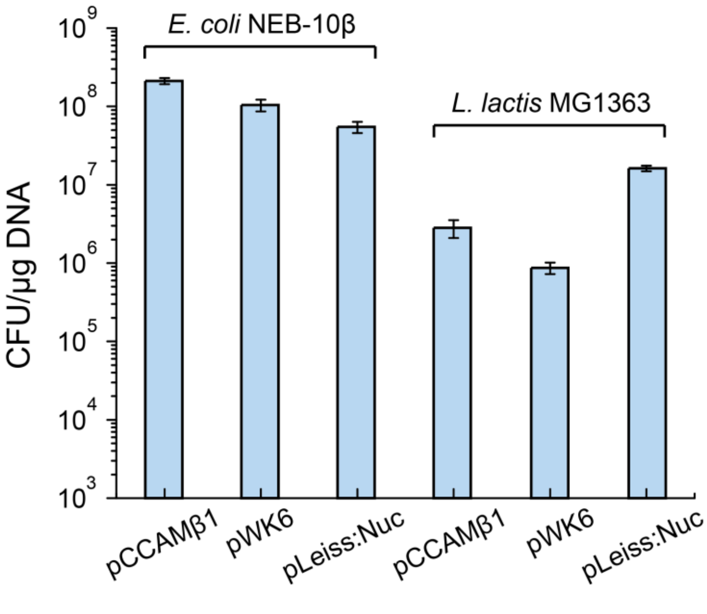

Figure S1. Transformation efficiencies of PEVLAB plasmids in *E. coli* and *L. lactis*.

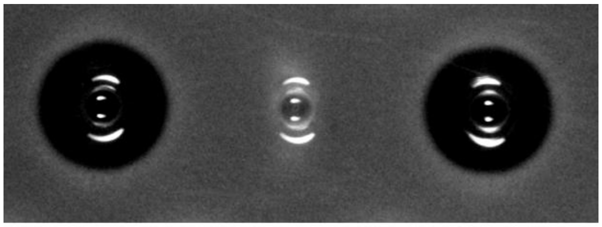

A B C

Figure S2. Confirmation of nisin production in the engineered strain. Characterization of nisin in the engineered strain through agar diffusion assay. Inhibition zones formed from: (a), standard nisin. (b), *L. lactis* MG1363/pCCAMβ1. (c), *L. lactis* MG1363/pWK6.

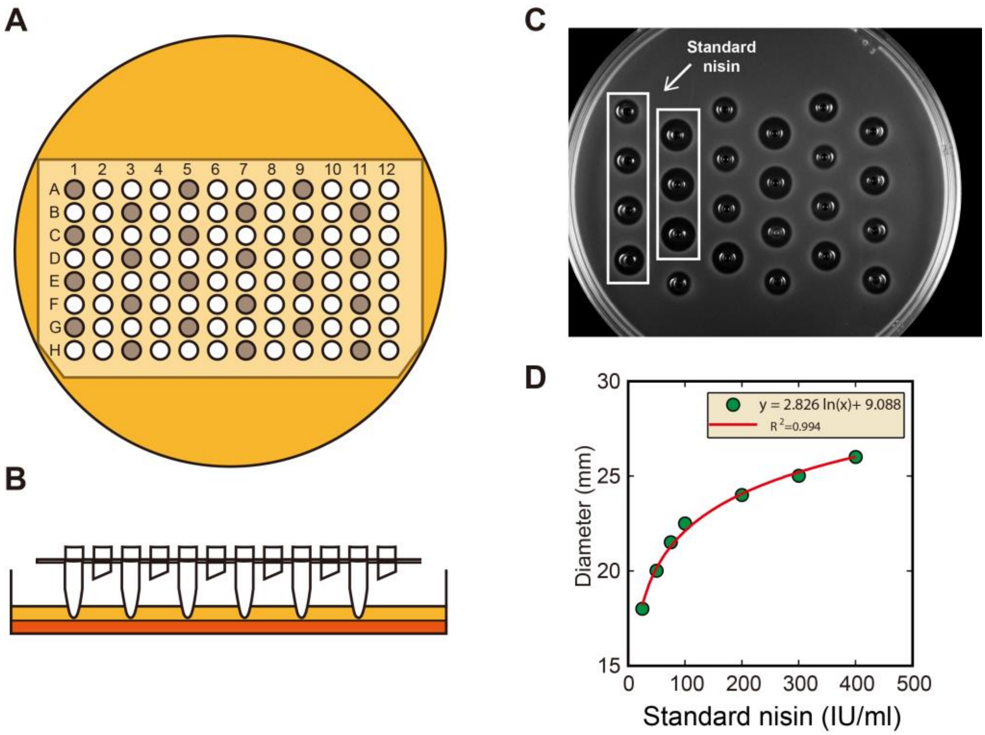

Figure S3. Schematic diagram of agar diffusion assay and standard curve. (**A-B**) Schematic illustrations of well molding using a PCR plate. Tubes in the PCR plate are selectively removed (the white ones) to provide enough space for individual inhibition zones. The bottom agar was seeded with the indicator strain *L. lactis* 117, and the wells were made in the top layer. (**A**): top view; (**B**): side view. (**C**) A typical agar diffusion assay. The wells in the white boxes were formed by a standard nisin solution with concentrations of 25 IU, 50 IU, 75 IU, 100 IU, 200 IU, 300 IU and 400 IU respectively from top to bottom and left to right. The remaining wells were from samples containing nisin. (**D**) A graphical representation of the standard nisin curve from the data collected in panel c. For an agar diffusion assay, every plate is loaded with standard nisin (white boxes) in order to provide a standard curve for calculating the nisin concentrations of the rest wells.

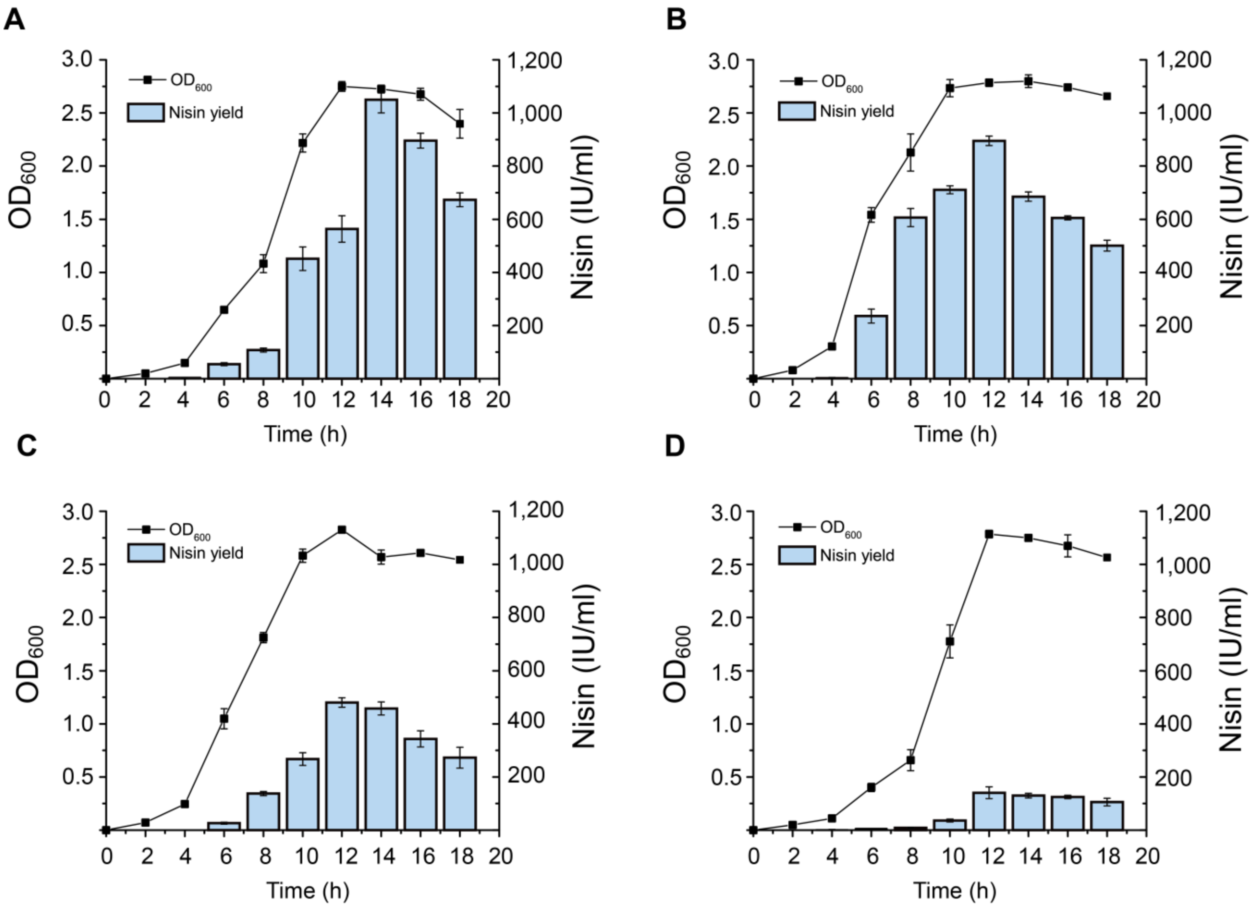

Figure S4. Representative temporal nisin production profiles of selected constructs from the heat map. The temporal profiles of the growth (line) and nisin production (column) of the constructs $A_{25607}B_{186955}$ (**A**), $A_{25607}B_{111924}$ (**B**), $A_{25607}B_{8228}$ (**C**) and $A_{25607}B_{2197}$ (**D**) were measured. Data were acquired from triplicate assays with the means and standard deviations illustrated in the figure. Additional discussion is available in Supplementary Discussion.

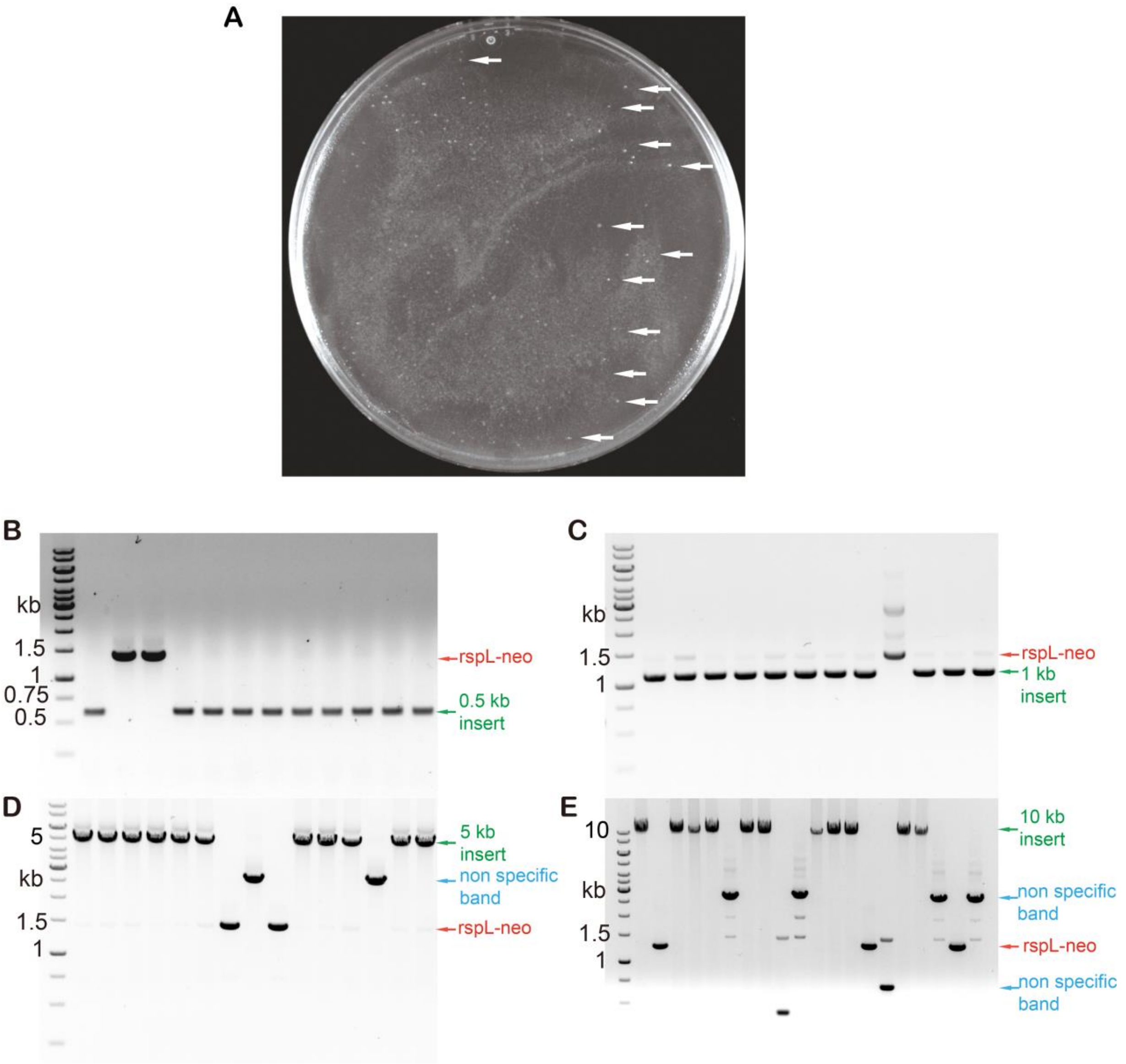

Figure S5. Counter-selection plate and PCR verification. (**A**) Colonies from a representative counter-selection plate. Arrows indicate the colonies on a thin bacterial lawn. (**B-E**) PCR verifications of colonies from different counter-selection experiments. Green arrows show correct inserts with a size of 0.5 kb, 1 kb, 5 kb and 10 kb. Red arrows show the negative results with the 1.5 kb band of the *rspL-neo* cassette. Blue arrows indicate the nonspecific recombination due to long inserts. The bands of 0.5 kb, 1 kb and 5 kb are directly from colony PCR. The 10 kb band is amplified from the plasmid template prepared from the colonies.

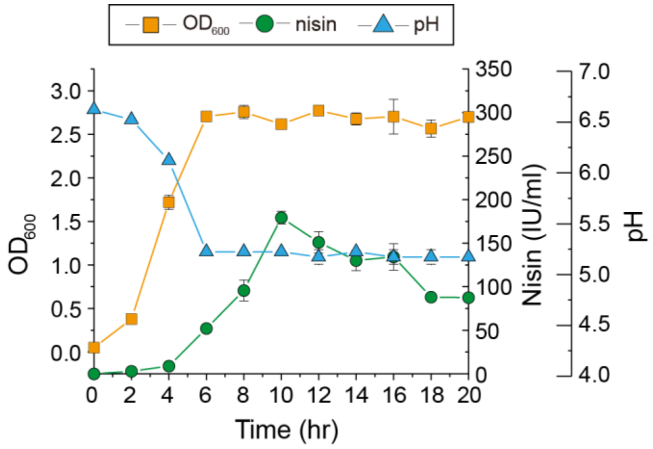

Figure S6. Nisin production profile of *L. lactis* MG1363/pWK6-lctA/F. Overnight cultures of *L. lactis* MG1363/pWK6-lctA/F were inoculated at a ratio of 1:50 into GM17 medium at initial pH of 6.6. Samples were taken every two hours to measure OD_{600}, nisin concentration in the supernatant, and pH value.

Table S1. Summary of primers used in this study

Name	Sequence (5' to 3')	description
**Construction of pCCAMβ1**		
PAMR1	CATTTTGAGGCATTTCAGTCAGTTGCTTCGGGAATTCTCATGTTTGACAGCTTATC	Amplify pAMβ1 origin+Erm
PAMF2	CGCGATCCGTAGCGGTTTTCAAAATTTGC	
PCCF	AAGCAACTGACTGAAATGCCTCAAAATG	Amplify pCC1BAC origin
PCCR2	GCAAATTTTGAAAACCGCTACGGATCGCGGCCGCGTCGACAGCGACACACTTGCATCGG	
**Construction of NEB10βmutS**		
MutSAmpF	ACTCGCCGTTCGCTTCTTCCCCTGAAATGATTAACTCCGGTAATAATGGTTTCTTAGACG	Amp primer for replacing MutS
MutSAmpR	TCGAAATAGTGGGTAGCAAATAACGTCAATGCCTTAATCTTATCAAAAAGGATCTTCACC	
MutSF	ACAGCATCTTTCCCGGAACCAGC	MutS knockout verifying and sequencing
MutSR	ACCATCCACTTGCGTAGCGG	
**Construction of pBeta**		
aadApucF	CGTTTGTTCGCCCAGCTTCTGT TTTAATTTAAAAGGATCTAGGTGAAG	Primers for pUC origin
lacOPpucR	CTCTGGTAAGGTTGGGAAGCCTCGGCTGCGGCGAGCGGTAT	
puclacOPF	ATACCGCTCGCCGCAGCCGA GGCTTCCCAACCTTACCAGAG	Primers for Plac+PlacIq+lacI
betalacOPR	CAGCGTTGCGAGTGCAGTACTCATAGCTGTTTCCTGTGTGAAATTGTTATC	
lacOPbetaF	GATAACAATTTCACACAGGAAACAGCTATGAGTACTGCACTCGCAACGCTG	Primers for beta
aadAbetaR	CCCTAGAGAGACGAAAGTGATTGCTCATGCTGCCACCTTCTGCTCT	
pucaadAF	CTTCACCTAGATCCTTTTAAATTAAA ACAGAAGCTGGGCGAACAAACG	Primers for aadA
betaaadAR	AGAGCAGAAGGTGGCAGCATGA GCAATCACTTTCGTCTCTCTAGGG	
**Construction of pWK6**		
Nisin 159	TCACGCCAATAACTTAGATTAAAATCACCGTCACC	Forward primer for nisin gene cluster
Nisin 14703	CAATACGAATCCATCAAAGTTTAGGGTATCTTACGCC	Reverse primer for nisin gene cluster

pccpamnisR	ACCCTAAACTTTGATGGATTCGTATTGCGGCCGCAGGAATGAATTACTATCCCTTTTATC	Forward primer for pCCAMβ1
pccpamnisF	TGATTTTAATCTAAGTTATTGGCGTGAGCGGCCGCGACAGCGACACACTTGCATCGGATG	Reverse primer for pCCAMβ1
**Selection primers for stem-loop site**		
loopFSmKn	AAATAACCAAATCAAAGGATAGTATTTTGTTAGTTCAGACATGGCCTGGTGATGATGGCG	Forward primer for neo-rspL
loopRSmKn	GAGCTTTAAATGAACTTTTTATCATGTTTTTTCCTCTCTCAGAAGAACTCGTCAAGAAGG	Reverse primer for neo-rspL
**Verification, PCR and sequencing primers for stem-loop site**		
loop-LF	CGTAAGCAAATAACCAAATCAAAG	Upstream primer
Loop-RR	TATTTCTTACTAAAAACGGTTGAGC	Downstream primer
nisBRseq	TCATAGAGTTTAGGATTAGC	Sequencing primer
**Primers to amplify 0.5 kb-10 kb non-selectable DNA for counter-selection**		
loopF321F	AAATAACCAAATCAAAGGATAGTATTTTGTTAGTTCAGACATCTTGTCAGACGAGGTGCT	Forward primer for all fragments
loopR820R	GGTTGAGCTTTAAATGAACTTTTTATCATGTTTTTTCCTCTCGTAATGGTAATCCTGGTA	Reverse primer for 0.5 kb
loopR1324R	GGTTGAGCTTTAAATGAACTTTTTATCATGTTTTTTCCTCTCTGATGGGTCTTAAAGGTG	Reverse primer for 1 kb
loopR2368R	GGTTGAGCTTTAAATGAACTTTTTATCATGTTTTTTCCTCTCGTAAACCAGGGACAGACG	Reverse primer for 2 kb
loopR5210R	GGTTGAGCTTTAAATGAACTTTTTATCATGTTTTTTCCTCTCTTATGGCGGTCAGTTGTG	Reverse primer for 5 kb
5404R	GGACAAGAACTAAATAATGGAGGAC	Helper primer for 10 kb assembly
5312F	TTGAATCGTAAAATATCCAGTGGCTG	
loopR10071R	GGTTGAGCTTTAAATGAACTTTTTATCATGTTTTTTCCTCTCAAGAAGCAATAGGGAAGC	Reverse primer for 10 kb
looppucF	AACATGATAAAAAGTTCATTTAAAGCTCAACCTATCCGCTCACAATTCCACACAAC	pUC19 primer for 10 kb assembly
looppucR	CTAACAAAATACTATCCTTTGATTTGGTTATTTGTATTTTCTCCTTACGCATCTGTG	
**Primers to refactor nisin promoters**		
PnisAknsmF	GATTTTCTAGTTCCTGAATAATATAGAGATAGGTTTATTGAGGGCCTGGTGATGATGGCG	PnisA to KnStrp

PnisAKnsmR	GTACTCATTTTGAGTGCCTCCTTATAATTTATTTTGTA TCAGAAGAACTCGTCAAGAAGG	
AproF	GATTTTCTAGTTCCTGAATAATATAGAGATAGGTTTATTGAG TTGACATTTGTTACTATC	PnisA counter selection with PlctA
AproR	GTACTCATTTTGAGTGCCTCCTTATAATTTATTTTGTA GTATTTCCTTACCTTCGAAATC	
nisP3KnsmF	ATCCTTGGTATATTGAAAAGAAAGACTAAAAATTGATAGATT GGCCTGGTGATGATGGCG	nisRK deletion and PnisF to KnStrp
nisF5KnsmR	TTGAATTTTTACCTGCATTATATTTCCTCACTTTATTT TCAGAAGAACTCGTCAAGAAGG	
F proF	ATCCTTGGTATATTGAAAAGAAAGACTAAAAATTGATAGATT CATCATTTGGATGTCTTG	PnisF counter selection
FproR	TTGAATTTTTACCTGCATTATATTTCCTCACTTTATTT CTTTTTGATAAATTTAAAACAG	

Table S2. Sequences of ssDNA oligos used for SPE in this study

Name	Sequence[a] (5'-3')	Notes
nisAN6	T*A*CCAAATCCAAGTTAAAATCTTTTGTACTCATTTTGAGTGC NNNNNNATAATTTATTTTGTAGTTCCTTCGAACGAAATCATTG TATCT	Test efficiency of ssDNA recombination.
nisA8-5	T*A*CCAAATCCAAGTTAAAATCTTTTGTACTCATTTTGAGTGC STCSSTATAATTTATTTTGTAGTTCCTTCGAACGAAATCATTGT ATCT	Degenerate oligo for generating nisA library.
nisB12-7	A*A*AACGGTTGAGCTTTAAATGAACTTTTTATCATGTTTTTTHC TWCCKTTATTTTTATAAGCTATTTAGCAACCCTAAATAACTTAT AAA	Degenerate oligo for generating nisB library.
nisA1	C*C*AAATCCAAGTTAAAATCTTTTGTACTCATTTTGAGTGCCT CCTTATAATTTATTTTGTAGTTCCTTCGAACGAAATCATTGTAT CTAA	Test efficiency of point mutation of ssDNA recombination.
A25607	T*A*CCAAATCCAAGTTAAAATCTTTTGTACTCATTTTGAGGTC CTTCCGTTTATTTATTTTGTAGTTCCTTCGAACGAAATCATTGT ATCT	Oligo for constructing nisA library
A18134	T*A*CCAAATCCAAGTTAAAATCTTTTGTACTCATTTTGAGTGG CCCCTCTCTATTTATTTTGTAGTTCCTTCGAACGAAATCATTGT ATCT	Oligo for constructing nisA library
B186955	A*A*ACGGTTGAGCTTTAAATGAACTTTTTATCATGTTTTTGCCT CCTTATACTTTTATAAGCTATTTAGCAACCCTAAATAACTTATA AAA	Oligo for constructing nisB library

a, The phosphorothioated DNA bases are labeled with symbol "*" .

Table S3. RBS sequences and their theoretical translation initiation rates

Name	RBS Sequence (From 5' to 3')	Theoretical translation Initiation Rate (AU)
**nisA**		
nisA wild type	GGAACTACAAAATAAATTATAAGGAGGCACTCAAA	143745
nisA7[a]	**GGAACTACAAAATAAATTATASSGASGCACTCAAA**	RBS library, see below
	GGAACTACAAAATAAATTATAGGGAGGCACTCAAA	125591
	GGAACTACAAAATAAATTATACGGAGGCACTCAAA	46662
	GGAACTACAAAATAAATTATAGCGAGGCACTCAAA	13843
	GGAACTACAAAATAAATTATAGGGACGCACTCAAA	9233
	GGAACTACAAAATAAATTATACGGACGCACTCAAA	3430
	GGAACTACAAAATAAATTATACCGAGGCACTCAAA	2237
	GGAACTACAAAATAAATTATAGCGACGCACTCAAA	1275
	GGAACTACAAAATAAATTATACCGACGCACTCAAA	294
A25607	GGAACTACAAAATAAATAAACGGAAGGACCTCAAA	25607
A18134	GGAACTACAAAATAAATAGAGAGGGGCCACTCAAA	18134
**nisB**		
nisB wild type	CTAAATAGCTTATAAAAATAAAGAGAGGAAAAAAC	104148
nisB12[a]	**CTAAATAGCTTATAAAAATAAMGGWAGDAAAAAAC**	RBS library, see below
	CTAAATAGCTTATAAAAATAAAGGAAGGAAAAAAC	111924
	CTAAATAGCTTATAAAAATAAAGGAAGTAAAAAAC	63941
	CTAAATAGCTTATAAAAATAAAGGTAGGAAAAAAC	54475
	CTAAATAGCTTATAAAAATAAAGGAAGAAAAAAAC	24407
	CTAAATAGCTTATAAAAATAAAGGTAGTAAAAAAC	19932
	CTAAATAGCTTATAAAAATAAAGGTAGAAAAAAAC	8228
	CTAAATAGCTTATAAAAATAACGGAAGGAAAAAAC	4508
	CTAAATAGCTTATAAAAATAACGGAAGTAAAAAAC	2197
	CTAAATAGCTTATAAAAATAACGGTAGGAAAAAAC	1781
	CTAAATAGCTTATAAAAATAACGGAAGAAAAAAAC	798
	CTAAATAGCTTATAAAAATAACGGTAGTAAAAAAC	652

	CTAAATAGCTTATAAAAATAACGGTAGAAAAAAAC	269
B18	CTAAATAGCTTATAAAAGTATAAGGAGGCAAAAAC	186955

a. The red bold sequences are degenerate RBS sequences. The red regular sequences below indicate the RBS library generated from the degenerate RBS sequence.

Table S4. Mutants with higher nisin productivity generated by modifying RBS of nisA and nisB

**Theoretical translation initiation rates of nisA and nisB**	**Nisin productivity (IU/ml)**
$A_{25607}B_{186955}$	1023.5
$A_{25607}B_{54475}$	909.8
$A_{46662}B_{186955}$	897.9
$A_{25607}B_{111924}$	895.2
$A_{125591}B_{186955}$	849.9
$A_{25607}B_{63941}$	841.7
$A_{18134}B_{186955}$	805.3
$A_{18134}B_{111924}$	800.2
$A_{18134}B_{63941}$	736.7
$A_{143745}B_{104148}$ (Wild type)	600.0

Reference

1. Wang, H.H., Isaacs, F.J., Carr, P.A., Sun, Z.Z., Xu, G., Forest, C.R. and Church, G.M. (2009) Programming cells by multiplex genome engineering and accelerated evolution. *Nature*, **460**, 894-898.
2. Ellis, H.M., Yu, D. and DiTizio, T. (2001) High efficiency mutagenesis, repair, and engineering of chromosomal DNA using single-stranded oligonucleotides. *Proc. Natl. Acad. Sci. USA*, **98**, 6742-6746.
3. Bird, A.W., Erler, A., Fu, J., Hériché, J.-K., Maresca, M., Zhang, Y., Hyman, A.A. and Stewart, A.F. (2012) High-efficiency counterselection recombineering for site-directed mutagenesis in bacterial artificial chromosomes. *Nat. Methods*, **9**, 103-109.
4. Holo, H. and Nes, I.F. (1989) High-frequency transformation, by electroporation, of *Lactococcus lactis* subsp. *cremoris* grown with glycine in osmotically stabilized media. *Appl. Environ. Microbiol.*, **55**, 3119-3123.
5. Kong, W., Kong, J., Hu, S., Lu, W., Wang, K. and Ji, M. (2011) Enhanced expression of PCV2 capsid protein in *Escherichia coli* and *Lactococcus lactis* by codon optimization. *World J. Microbiol. Biotechnol.*, **27**, 651-657.
6. Vuyst, L.d. (1995) Nutritional factors affecting nisin production by *Lactococcus lactis* subsp. *lactis* NIZO 22186 in a synthetic medium. *J. Appl. Bacteriol.*, **78**, 28-33.
7. Pongtharangkul, T. and Demirci, A. (2006) Evaluation of Culture Medium for Nisin Production in a Repeated ‐ Batch Biofilm Reactor. *Biotechnol. Prog.*, **22**, 217-224.
8. Liu, X., Chung, Y.-K., Yang, S.-T. and Yousef, A.E. (2005) Continuous nisin production in laboratory media and whey permeate by immobilized *Lactococcus lactis*. *Process Biochem.*, **40**, 13-24.
9. de Arauz, L.J., Jozala, A.F., Mazzola, P.G. and Vessoni Penna, T.C. (2009) Nisin biotechnological production and application: a review. *Trends Food Sci. Technol.*, **20**, 146-154.
10. De Vuyst, L. and Vandamme, E.J. (1992) Influence of the carbon source on nisin production in *Lactococcus lactis* subsp. *lactis* batch fermentations. *J Gen. Microbiol.*, **138**, 571-578.
